# *Vibrio cholerae* Skin and Soft Tissue Infection Complicated by Sepsis in an Immunocompromised Patient: A Rare Case Report

**DOI:** 10.1002/ccr3.70086

**Published:** 2025-01-06

**Authors:** Erin Rebecca Carr, Adrian Estepa, Jordan Colson, Folusakin Ayoade

**Affiliations:** ^1^ University of Miami Miller School of Medicine Miami Florida USA; ^2^ Division of Infectious Diseases, Department of Medicine University of Miami Miller School of Medicine Miami Florida USA; ^3^ Department of Pathology and Laboratory Medicine University of Miami Miller School of Medicine Miami Florida USA

**Keywords:** bacteremia, cellulitis, sepsis, *Vibrio cholerae*

## Abstract

Physicians should consider non‐O1, non‐O139 *Vibrio cholerae* (NOVC) in the differential diagnosis of cellulitis complicated by sepsis, especially in immunocompromised patients when potential exposure exists. Due to the pathogen's potential for severe infections and rising incidence from environmental changes, we emphasize the need for increased awareness and appropriate treatment guidelines.

## Graphical abstract

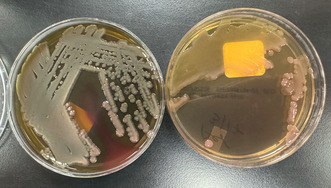

## 1 Introduction

*Vibrio cholerae (V. cholerae)* is a Gram‐negative, curved rod‐shaped bacterium found in various aquatic environments, including freshwater, estuarine, and marine ecosystems [1, 2]. It is the causative agent of cholera, a severe diarrheal disease that can lead to rapid dehydration and, if left untreated, death. Although over 200 serotypes of *V. cholerae* have been identified on the basis of their distinctive lipopolysaccharide O‐antigens, only 12 are currently recognized as pathogenic to humans [1]. Among these, the O1 and O139 serogroups are most notable, as they secrete cholera toxin (CT) and are responsible for the majority of cholera outbreaks [3]. Although the medical literature is limited, most case reports find extraintestinal infections to be more common with non‐O1, non‐O139 serotypes, and less so with the toxigenic O1 and O139 strains [1, 4, 5, 6, 7].

In contrast, non‐O1, non‐O139 *Vibrio cholerae* (NOVC) strains, although not typically associated with cholera epidemics, can be clinically significant. Although these strains do not produce CT and are infrequently implicated in widespread outbreaks, NOVC can be associated with cases of self‐limited gastroenteritis and extraintestinal infections, including bacteremia, meningitis, pneumonia, peritonitis, cholecystitis, salpingitis, otitis, and skin and soft tissue infections (SSTI) [1]. Various manifestations of SSTI have been documented in the medical literature, ranging from localized cellulitis, sometimes with hemorrhage and bullae, to the rare occurrence of necrotizing fasciitis [1]. Furthermore, emerging case report data seem to suggest higher vulnerability to NOVC infections in the immunocompromised and in those with specific comorbidities, such as liver disease, peripheral vascular disease, or diabetes mellitus [8, 9, 10, 11, 12].

The first documented case of NOVC infection in the United States was reported in 1974 [4]. Since then, over 350 cases have been documented worldwide up to 2019 [4]. These cases highlight the pathogenic potential of NOVC. Here, we report a case involving a Russian patient with underlying liver disease who developed a NOVC SSTI complicated by bacteremia, after swimming in the ocean off the coast of Miami Beach, Florida.

## 2 Case History/Examination

A 58‐year‐old male with past medical history significant for untreated hepatitis C virus (HCV) infection, alcoholic cirrhosis, hepatocellular carcinoma (HCC) status post‐radioembolization, atrial fibrillation, and heart failure with an improved ejection fraction, presented to the emergency department with a 2‐day history of left lower extremity pain.

The patient traveled from Russia to Miami for vacation and reported that his symptoms began approximately 36 h after a visit to South Beach (coastal Miami, Florida), where he swam in the ocean. Initially, he noticed pain and erythema in the left lateral calf, which he attributed to a sunburn. However, the pain progressively worsened, and he began to experience subjective fever, chills, and paresthesia. Of note, he denied any gastrointestinal symptoms. Eventually, he was unable to bear weight on his left leg, prompting him to seek medical attention. He denied any trauma, insect bites, or any abrasions to the left lower extremity related to his swimming experience. He also denied any consumption of raw or undercooked seafood and fish.

**FIGURE 1:**
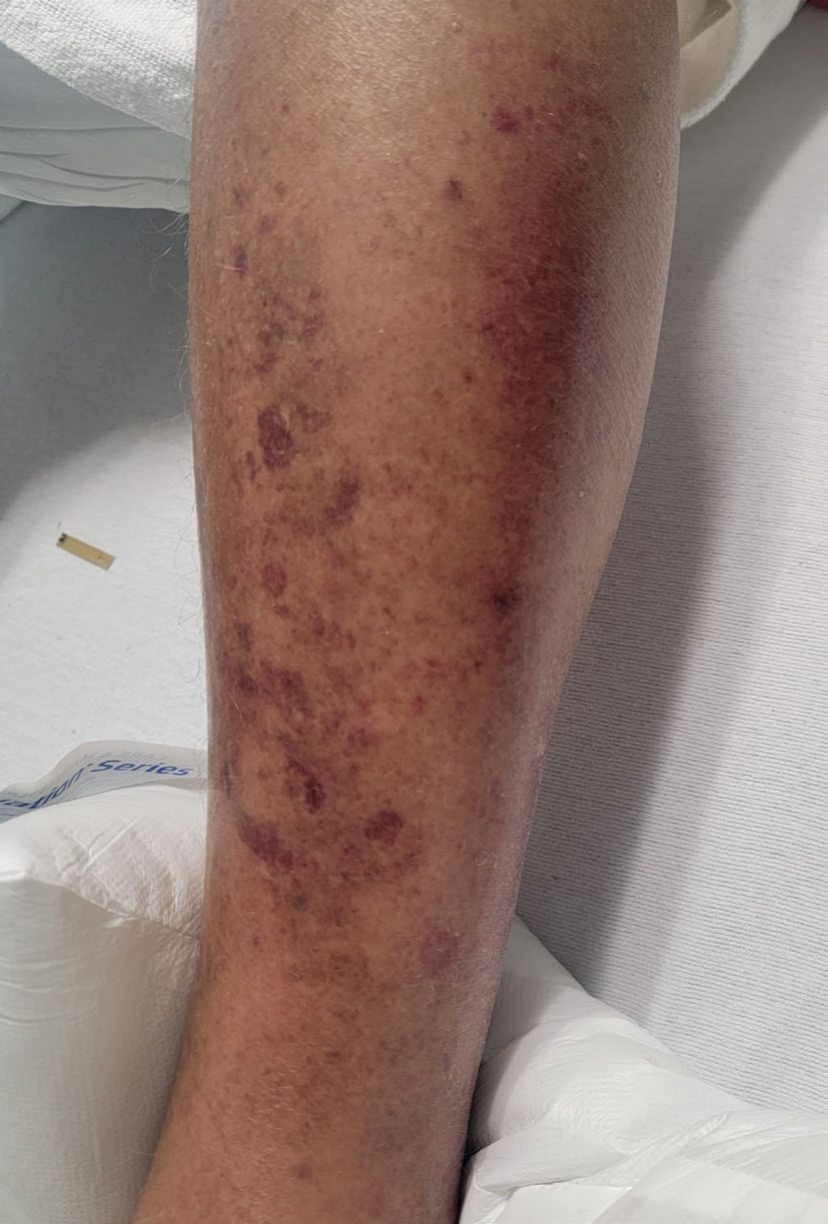
Left lateral calf with erythema, petechiae, and edema without bullae.

**FIGURE 2:**
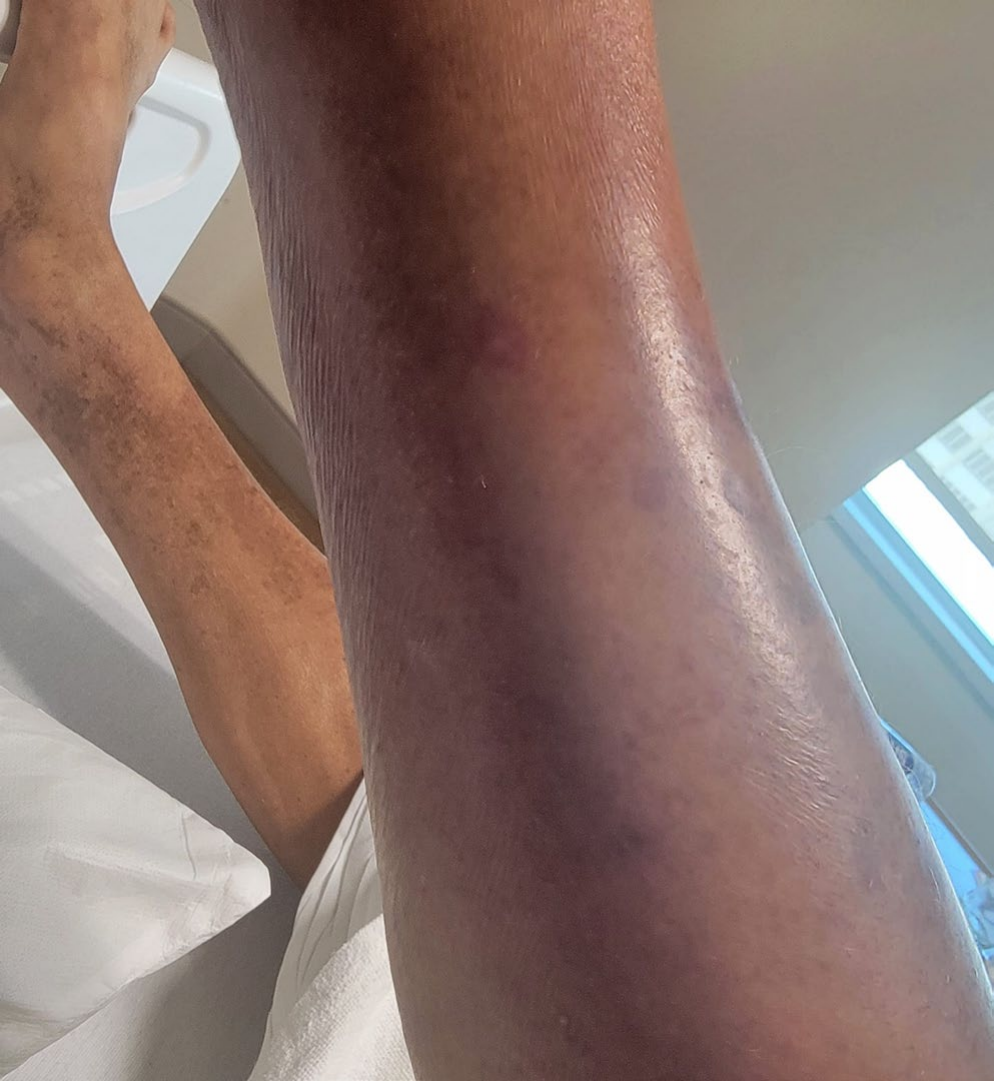
Left lateral calf with erythema, petechiae, and edema without bullae.

Upon arrival, the patient was febrile to 38.4°C, tachycardic to 117 bpm, and hypertensive to 160/101 mmHg. The physical examination revealed erythema, petechiae, and edema of the lateral left calf, which did not extend below the ankle or above the knee. The skin of the affected area on the left lower extremity was noticeably warmer compared with the right lower extremity. No crepitus, fluctuance, or bullae were present (Figures 1 and 2).

## 3 Methods

An X‐ray of the left tibia and fibula was unremarkable, with no evidence of acute fracture or dislocation. Laboratory findings revealed a white blood cell count of 9.5 × 10^3^/μL with both a relative neutrophilia of 88.5% and an absolute neutrophil count of 8350 cells/μL. Additionally, the patient had thrombocytopenia, likely secondary to his underlying liver disease, with a platelet count of 34 × 10^3^/μL. Due to concerns for cellulitis, the patient was empirically started on intravenous (IV) ceftriaxone 2 g every 24 h and IV vancomycin 15 mg/kg for methicillin‐resistant *Staphylococcus aureus* (MRSA) coverage, given the systemic signs of infection. Though no bullae were appreciated on physical examination, IV doxycycline 100 mg q12h was also initiated for *Vibrio* coverage because of the patient's exposure to ocean saltwater and underlying liver disease as a risk factor. Though exposure to water broadens the differential diagnosis of potential pathogenic organisms to include *Aeromonas hydrophilia*, nontuberculous *Mycobacterium*, including *M. fortuitum* and *M. marinum* , *Pseudomonas aeruginosa* , and *Vibrio* spp., it is crucial not to discount *Vibrio cholerae* [2, 13]. This is particularly important when gastrointestinal symptoms are absent, as evidenced in our case where the patient presented only with cutaneous involvement.

## 4 Results

**FIGURE 3:**
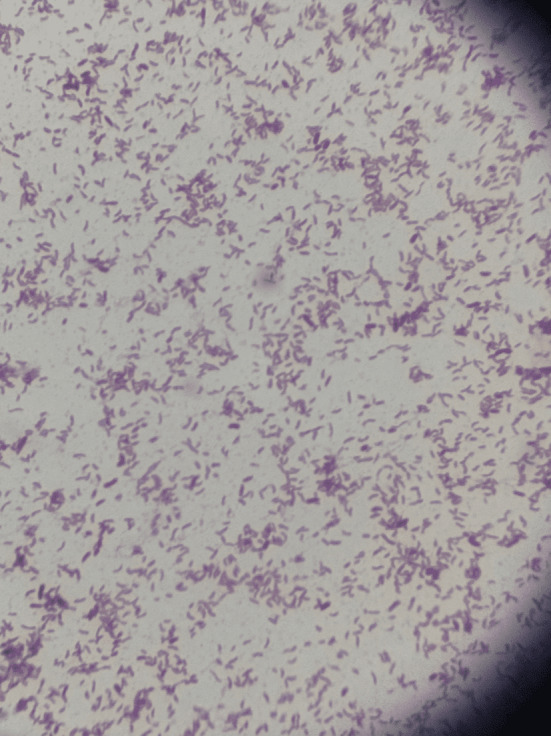
Gram stain showing curved Gram‐negative rods.

**FIGURE 4:**
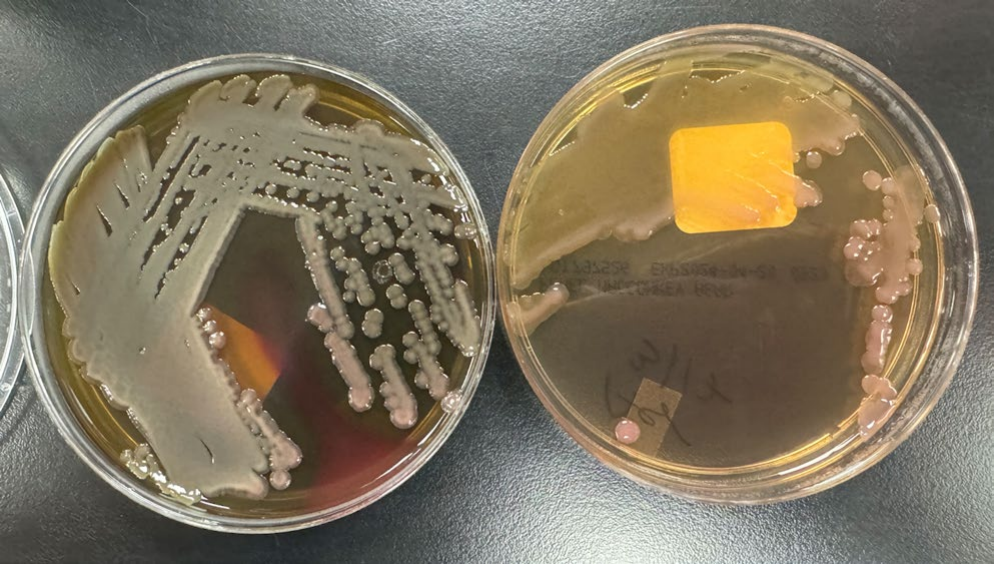
Blood agar (BAP) on the left demonstrating mucoid beta‐hemolytic colonies. Growth on MacConkey agar (MAC) on the right demonstrating lactose non‐fermenting isolate.

Blood cultures were subsequently obtained, and microbiological testing revealed beta‐hemolytic, mucoid lactose non‐fermenting gram‐negative rods (Figures 3 and 4). The BioFire Blood culture identification panel 2 (BCID2) was negative, but Matrix Assisted Laser Desorption/Ionization (MALDI) and Vitek identified *Vibrio cholerae* . Susceptibility testing was performed using Clinical and Laboratory Standards M45 standards and revealed susceptibilities to ampicillin, azithromycin, trimethoprim/sulfamethoxazole, and tetracycline. The patient had repeat blood cultures 2 days into his admission, which demonstrated clearance of *V. cholerae* bacteremia. Vancomycin and ceftriaxone were discontinued, and the patient was discharged on doxycycline 100 mg orally twice daily to complete a 14‐day course. Doxycycline was selected because of its better tolerability, more favorable side effect profile, high oral bioavailability, and supporting evidence for its use in *Vibrio* infections, as demonstrated by a meta‐analysis that favored tetracyclines over ampicillin, azithromycin, and TMP/SMX [14]. Additionally, TMP/SMX was avoided because of the patient's preexisting thrombocytopenia and its potential to exacerbate this condition. The patient completed his antibiotic course, leading to the resolution of his SSTI and sepsis.

The isolate was subcultured, sent to the Florida Department of Health and the US Centers for Disease Control and Prevention (CDC), and subsequently identified as non‐O1, non‐O139 via conventional serum agglutination.

## 5 Discussion

The patient's untreated HCV infection, alcoholic cirrhosis, and HCC significantly increased his susceptibility to NOVC infection. Although the reason for the high prevalence of *Vibrio* species (spp.) infection in cirrhotic patients is unclear, theories involving poor bactericidal activity, and impaired filtration function in the cirrhotic liver persist [15]. In cirrhotic patients, decreased phagocytic activity of the reticuloendothelial system and increased intestinal permeability may facilitate the passage of bacteria to the regional lymph nodes and systemic circulation, leading to the development of *V. cholerae* bacteremia [16]. It is also speculated that the hemolysin produced by certain strains of NOVC can contribute to invasive disease in immunocompromised hosts, due to its hemolytic property and ability to induce vacuolization [17]. Therefore, we recommend that patients at risk for NOVC infection, particularly those with liver disease, receive anticipatory guidance during their medical appointments. This should include advice on the dangers of consuming raw or undercooked seafood and fish, as well as cautions about the risks associated with exposure to fresh and saltwater, especially if they have any open wounds or abrasions [17].

Moreover, *Vibrio* spp. are increasingly recognized as important emerging pathogens worldwide. Although data remain mixed, this observation has been cited as attributable to multiple factors, including global rising water temperatures, plankton blooms, precipitation, and an increasing population with comorbidities associated with NOVC infections [18, 19, 20, 21, 22, 23, 24]. Events, such as tropical storms and resulting flash floods, which lead to standing water across regions like South Florida, may create conditions conducive to *Vibrio* proliferation. The heightened presence of *Vibrio* spp. in such environments underscores the necessity for timely recognition and management of these infections, especially given their potential for severe outcomes in immunocompromised individuals.

The clinical implications of *Vibrio* infections are particularly important for patients with cirrhosis, where the mortality rate can reach up to 75% in cases of concurrent skin infection [2]. This emphasizes the need for a low threshold of suspicion and highlights the urgency for developing specific treatment guidelines for NOVC SSTI and bacteremia, as no standard recommendations currently exist at the time of writing this case report. Although there have been no randomized clinical trials to establish a definitive treatment protocol and standard of care, retrospective surveillance analyses indicate higher mortality rates when beta‐lactam antibiotics are used alone compared with regimens that include fluoroquinolones alone, fluoroquinolones in combination with a beta‐lactam, or tetracyclines with a beta‐lactam [25]. This evidence ultimately guided the initial dual therapy approach for our patient, who was started on ceftriaxone and doxycycline to ensure comprehensive coverage against *V. cholerae* , and his clinical status significantly improved.

## 6 Conclusion

The patient's untreated HCV, alcoholic cirrhosis, and HCC significantly increased his risk of NOVC infection. Although NOVC infections carry a potentially high mortality rate in patients with liver disease, they are not well‐documented in the medical literature, and no treatment guidelines currently exist for NOVC SSTI and bacteremia. Environmental factors, such as rising water temperatures and increasing incidence of relevant comorbidities, may explain the increased incidence of NOVC infections. In the setting of exposure to fresh or brackish waters, health providers should consider non‐O1, non‐O139 *Vibrio cholerae* (NOVC) in the differential diagnosis of cellulitis complicated by sepsis, especially in immunocompromised patients, including those with liver cirrhosis.

## Author Contributions

**Erin Rebecca Carr:** writing – original draft, writing – review and editing. **Adrian Estepa:** writing – original draft, writing – review and editing. **Jordan Colson:** investigation, resources, writing – review and editing. **Folusakin Ayoade:** supervision, writing – review and editing.

## Ethics Statement

The authors confirm that the ethical policies of the journal, as noted on the journal's author guidelines page.

## Consent

Written consent was obtained from the patient for publication.

## Conflicts of Interest

The authors declare no conflicts of interest.

## Data Availability

All data underlying the results are available as part of the article and no additional source data are required.
